# Comparison of second and third-generation parathyroid hormone assays at a tertiary hospital in South Africa

**DOI:** 10.4102/ajlm.v14i1.2700

**Published:** 2025-08-30

**Authors:** Nokuthula Nhlapo, Doreen Jacob, Siyabonga Khoza, Mpho R. Maphayi

**Affiliations:** 1 Department of Chemical Pathology, Faculty of Health Sciences, University of the Witwatersrand, Johannesburg, South Africa; 2 Department of Chemical Pathology, National Health Laboratory Service, Johannesburg, South Africa

**Keywords:** parathyroid hormone, second-generation PTH assays (intact PTH), third-generation PTH assays (PTH 1–84), chronic kidney disease, dialysis, method comparison, bias, estimated glomerular filtration rate, hyperparathyroidism

## Abstract

**Background:**

Parathyroid hormone (PTH) measurement is key for diagnosing parathyroid disorders, and for management of chronic kidney disease. Available PTH assays include second (intact PTH) and third (PTH 1–84) generations. Data comparing interchangeable use are insufficient.

**Objective:**

The objective of this study was to compare intact and 1–84 PTH assays to determine the difference in analytical performance and impact on clinical interpretation.

**Methods:**

A method comparison was done on residual samples with PTH requests (06 April 2022 – 21 September 2022) from a tertiary hospital in South Africa. Parathyroid hormone was measured using both intact PTH and 1–84 PTH assays. Clinical performance was compared in the diagnosis of hypo- and hyperparathyroidism, and in pre-dialysis and dialysis chronic kidney disease patients.

**Results:**

Among 481 samples, intact PTH had a higher median concentration than PTH 1–84 (9.85 pmol/L vs. 8.51 pmol/L, *p* < 0.0001), but the two showed good correlation (*r* = 0.994, *p* < 0.0001). Regression analysis revealed systematic (intercept = 0.887 pmol/L [95% confidence interval: 0.788 – 1.005]) and proportional differences (slope = 0.713 pmol/L, [95% confidence interval: 0.703 – 0.723]), with increased deviations at higher concentrations. The average bias was 18.5%, exceeding allowable limits. Among the 276 patients (170 women, 106 men, age range: 18–89 years) included in the clinical study, interpretation was unchanged.

**Conclusion:**

A bias was observed between the PTH assays, indicating that they should not be used interchangeably. However, no changes in clinical interpretation were observed when one assay was used over the other.

**What this study adds:**

The study confirms the recommendation by Kidney Disease: Improving Global Outcomes for the use of assay-specific upper limit of normal instead of generic cut-off in dialysis patients. This study further highlights the need for standardisation of PTH assays.

## Introduction

Chronic kidney disease (CKD) is a global health problem with a prevalence of 5% – 10%.^1^ In Africa, CKD presents a major public health concern, particularly against a background of limited resources.^2^ Such patients have an increased risk of CKD-mineral and bone disorder (CKD-MBD), which is associated with high morbidity and mortality.^1^ Bone histomorphometry is the gold standard in the assessment of renal osteodystrophy, which reflects the skeletal facet of CKD-MBD. However, it is invasive, expensive, and requires expertise not readily available, thus measurement of circulating biomarkers, including parathyroid hormone (PTH), offers an attractive non-invasive alternative.^1,3^ Changes in PTH concentrations contribute to the bone abnormalities observed in CKD-MBD.^4^ This makes its measurement essential in the management of CKD-MBD.

Parathyroid hormone regulates calcium and phosphate homeostasis and bone remodelling,^4^ and determination thereof is crucial in the diagnostic workup of hypocalcaemia, hypercalcaemia, and in the perioperative assessment in thyroid or parathyroid surgery.^1,4^ Therefore, accurate and reliable PTH assays are needed for proper patient management. Parathyroid hormone is composed of a single polypeptide produced exclusively by the chief cells of parathyroid glands, with a molecular weight of ~9.5 kDa.^5^ It is sequentially cleaved at the amino terminal end to yield the mature biologically active 84 amino acid molecule PTH (1–84), which consists of two main parts: the amino (N) terminus, made up of the first 34 amino acids, and the carboxyl (C) terminus, that contains the remaining 50 amino acids. As a result of rapid proteolytic cleavage in circulation, PTH exists as intact PTH (1–84) and various fragments, that is, N-terminal, mid-region and C-terminal PTH, the most abundant being 7–84 PTH.^6^ Although the plasma half-life of PTH (1–84) is between 2 and 4 min, C-terminal PTH fragments have a longer half-life of several hours because of renal clearance.^6,7^ The latter may account for more than 20% of circulating PTH in normal kidney function individuals and up to 45% in patients with renal failure.^6^ This heterogeneity of PTH in circulation has made its measurement challenging.

Parathyroid hormone measurements have numerous pitfalls. Currently, no reference method exists, and although the use of a single recognised international standard, for example, World Health Organization PTH 95/646, has been proposed, it has not been fully implemented, and PTH measurement remains unstandardised.^8,9^ The radioimmunoassay was a first-generation competitive PTH assay that utilised a single polyclonal antibody generated against epitopes in the mid- or C-terminus of the PTH peptide.^7^ The radioimmunoassays had several limitations, including cross reactivity with the biologically inactive C-fragments, which are highest in CKD patients because of decreased renal clearance.^10,11^ To overcome this issue, the immunometric second- and third-generation PTH assays were developed. The second-generation PTH assay is a non-competitive sandwich immunoassay that makes use of two antibodies, generated against the mid-region N-terminal and C-terminal amino acids. It was assumed that it detected the biologically active PTH (1–84) and avoided cross reactivity with C-fragments, and thus was referred to as the ‘intact’ PTH assay.^11,12^ However, subsequent studies revealed that these assays overestimated values in patients with kidney failure compared to PTH values in normal subjects because of the accumulation of N-truncated PTH fragments, the most abundant of which is 7–84 PTH.^13,14^ The third-generation PTH assays use a capture antibody against the C-terminal amino acids similar to the second-generation. However, the detection antibody is directed against epitopes in the first four amino acids of the N-terminus, which avoids cross reactivity with 7–84 PTH fragments.^7,11^ Third-generation assays were termed bio-intact (bio-PTH) or whole PTH assays, as they were more specific for PTH (1–84).^12^

Second-generation assays have provided the basis for the Kidney Disease: Improving Global Outcomes (KDIGO) clinical guidelines on management of CKD-MBD.^12,15^ Significant inter-method variability among second-generation assays has been shown, thus affecting comparability of results.^3^ Taking this into consideration, the recent KDIGO guidelines proposed utilisation of multiples of 2–9-fold of the upper limit of normal for a given PTH assay, instead of absolute values in CKD patients on dialysis. In patients not on dialysis, the KDIGO guidelines state that the target PTH concentration is unknown and thus recommend monitoring trends rather than a single value.^15^

Research has shown that third-generation assays produce results that are 50% – 60% lower in CKD patients, and 15% lower in non-CKD patients, when compared to second-generation assays.^10,12^ Third-generation assays are more specific to bioactive PTH than second-generation, and should in theory be more accurate in the evaluation of PTH status. Yet, there is still dispute as to which assay is preferable and whether third-generation assays offer improved clinical value.^5^ Second-generation assays are readily available, extensively researched and form part of current clinical guidelines.^12^ In contrast, there is insufficient research for the third-generation to be universally adopted in clinical practice.^12^

In our setting, the National Health Laboratory Service services two major academic hospitals, namely Chris Hani Baragwanath Academic Hospital and Charlotte Maxeke Johannesburg Academic Hospital (CMJAH), which utilise the second- and third-generation PTH assays, respectively, from the same manufacturer with similar reference intervals. There is considerable movement of patients and blood samples between the two hospitals, which may affect PTH interpretation and clinical decision-making depending on the assay used. The aim of this study was to compare the analytical performance of second- and third-generation PTH assays and their impact on clinical interpretation of hypo- and hyperparathyroidism, and in pre-dialysis and dialysis CKD based on current KDIGO guidelines.

## Methods

### Ethical considerations

This study has been approved by the Human Research Ethics Committee (Medical) of the University of the Witwatersrand and performed in line with the Declaration of Helsinki. The study made use of remnant patient samples on which routine PTH measurement was requested. Ethics clearance (clearance certificate number M210475) was obtained for use of remnant samples and informed consent was not required. All patient data obtained were fully anonymised and de-identified before accession.

### Study design

This cross-sectional study was conducted between 06 April 2022 and 21 September 2022, in two parts. The first part was a method comparison study conducted on residual patient samples from both in- and outpatients on which a PTH was requested at CMJAH National Health Laboratory Service. These samples were tested on both second- and third-generation PTH assays consecutively. The second part assessed clinical interpretation of the assays’ results among a subset of the same group of patients, using data from the laboratory information system.

### Sample size calculations

For a method comparison study, a minimum of 40 samples is required to achieve statistical significance as per Clinical Laboratory Standard Institute EP09-A3 guideline.^16^ However, more than 100 are recommended to improve statistical power. All PTH samples received during the study period were considered for the study and included if they met the criteria.

For clinical interpretation, the Equation 1^17^ was used to derive the sample size (*n*):

$$n=\frac{zp(1-p)}{d^{2}}$$ [Eqn 1]

utilising a prevalence of 6.4% in South Africa for CKD; *z* denotes the *z*-score of 1.96 that corresponds to the confidence interval (CI) of 95%, *p* is expected prevalence (6.4%) and *d* is precision, which reflects the effect size and was estimated to be 5%.^17,18^ A minimum of 92 samples from CKD patients were required to demonstrate a difference between the two assays.

### Method comparison

Samples were collected in BD Vacutainer® ethylenediamine-tetraacetic acid tubes™ (Becton Dickinson and Company, Franklin Lakes, New Jersey, United States) by clinicians as part of the routine clinical care. Samples of sufficient volume and within recommended stability period of 48 h when stored at 2 °C – 8 °C were included. The samples were initially analysed using the third-generation Roche TH 1–84 assay on the Roche cobas^®^ e602 analyser (Roche Diagnostics, Mannheim, Germany), the routine assay at CMJAH. The second-generation intact PTH assay was validated on the same analyser for the study at CMJAH laboratory, replicating the assay used at Chris Hani Baragwanath Academic Hospital. Parathyroid hormone is unstable in blood samples; to maintain sample stability, method comparison was performed at the same site. After analysing the samples on the third-generation PTH assay, they were immediately re-analysed using the newly validated second-generation Roche intact PTH assay.

### Laboratory analysis

A verification study for the second-generation PTH assay was performed according to the Clinical Laboratory Standard Institute EP15-A3 guideline.^19^ This was done because the second-generation PTH assay was not validated on the Roche cobas^®^ e602 analyser at CMJAH. Assay performance was within the acceptable limits of the manufacturer’s claims with intra-assay variability of 0.9%, and inter-assay variability of 2.2%.

The PTH assays’ analytical performance was compared as per the Clinical Laboratory Standard Institute EP 09-A3 guideline.^16^ Analyses of samples for PTH were performed on an e602 module of a cobas^®^ 8000 system (Roche Diagnostics, Mannheim, Germany). The analytical characteristics of the PTH assays used are depicted in Online Supplementary Table 1.^20,21^ Biochemical parameters including creatinine, total calcium and phosphate were measured using standard chemistry methods on the Roche cobas^®^ 8000 analyser e702 module.

**TABLE 1:** Demographic and biochemical data for the patient samples used in clinical interpretation, Johannesburg, South Africa, 06 April 2022 to 21 September 2022.

Parameters	Overall (*N* = 276)				CKD (*n* = 146)				Non-CKD (*n* = 130)			
	Median	IQR	*n*	%	Median	IQR	*n*	%	Median	IQR	*n*	%
**Age (years)**	52.50	49.00–54.00	-	-	46.90	44.61–49.19	-	-	55.00	54.00–59.00	-	-
**Sex**												
Female	-	-	170	62	-	-	80	55	-	-	90	69
Male	-	-	106	38	-	-	66	45	-	-	40	31
**Parathyroid hormone (pmol/L)**												
Intact PTH	12.33	10.52–16.96	-	-	26.28	20.84–36.28	-	-	6.75	5.68–8.10	-	-
PTH 1–84	10.36	9.10–12.81	-	-	19.95	15.50–27.29	-	-	6.05	5.00–7.04	-	-
**Serum calcium (mmol/L)**	2.30	2.27–2.32	-	-	2.26	2.20–2.30	-	-	2.36	2.31–2.41	-	-
**Phosphate (mmol/L)**	1.26	1.17–1.36	-	-	1.30	1.19–1.42	-	-	1.16	1.06–1.33	-	-
**Creatinine (µmol/L)**	306.00	264.55–417.04	-	-	599.00	428.03–697.86	-	-	90.00	82.08–107.93	-	-
**eGFR (mL/min/1.73 m^2^)**	16.00	12.00–22.45	-	-	8.00	6.00–10.00	-	-	66.00	52.23–74.92	-	-
CKD Stage 1	-	-	3	-	93.00†	-	-	-	-	-	-	-
CKD Stage 2	-	-	9	-	65.00	58.41–81.03	-	-	-	-	-	-
CKD Stage 3a	-	-	9	-	52.00	45.28–56.00	-	-	-	-	-	-
CKD Stage 3b	-	-	9	-	36.00	33.14–37.86	-	-	-	-	-	-
CKD Stage 4	-	-	18	-	19.00	16.00–21.22	-	-	-	-	-	-
CKD Stage 5	-	-	70	-	5.50	5.00–6.00	-	-	-	-	-	-
CKD Stage 5D	-	-	28	-	5.50	4.36–7.00	-	-	-	-	-	-

*Source*: Adapted from Nhlapo N. Comparison of second and third generation parathyroid hormone (PTH) assays performed at Charlotte Maxeke Johannesburg Academic Hospital – Are they fit for purpose? [Master’s thesis]. Johannesburg: University of the Witwatersrand; 2023. Available from: https://hdl.handle.net/10539/44544

IQR, 25th and 75th interquartile range; CKD, chronic kidney disease diagnosed with > 3-month history of estimated glomerular filtration rate (eGFR) < 60 mL/min/1.73 m^2^. Non-CKD, patients without confirmed history of CKD.

† , sample size insufficient to calculate confidence interval.

### Clinical interpretation

The same data set used in the method comparison study was considered for the clinical performance study, and those that met criteria were included. The clinical performance of the two assays was assessed for their performance in the diagnosis of hypoparathyroidism and hyperparathyroidism and in CKD patients, including those on dialysis. Data were collected from the National Health Laboratory Service TrakCare^TM^ (InterSystems, Cambridge, Massachusetts, United States) laboratory information system during the study period (06 April 2022 and 21 September 2022) and included: age, gender, calcium, phosphate, creatinine, and 25 (OH) vitamin D. The glomerular filtration rate (GFR) was estimated using the Chronic Kidney Disease Epidemiology Collaboration 2009 equation without the race factor.^22^ Samples within storage stability limits, those with all required biochemical data and patients 18 years old and above were included for the clinical interpretation (Online Supplementary Figure 5b).

### Statistical analysis

The data obtained were captured onto Microsoft Excel^®^ (Microsoft, Seattle, Washington, United States) and analysed using MedCalc^®^, version 200218 (MedCalc Software, Ostend, Belgium). The Shapiro-Wilks test was used to assess for normality. Data were demonstrated to be not normally distributed and expressed as median and interquartile range. Categorical data were expressed as proportion and percentage. The Wilcoxon test was used to compare PTH concentrations between the two assays. A Mann-Whitney test was used to compare the median age between the CKD and non-CKD groups.

Intact PTH was used as the comparative method (X) and PTH 1–84 as the test method (Y). Passing-Bablok regression was used to assess method agreement, and the Bland-Altman difference plot was used to determine the magnitude of bias (observed differences) between the two methods. The observed inter-assay bias was compared with desirable analytical performance specifications (bias ±7.1%) for PTH derived from the European Federation of Clinical Chemistry and Laboratory Medicine biological variation database.^23^ The McNemar test was used to determine if there were significant differences in the classification of patients and if the clinical interpretation changes when one assay is used over the other. A *p*-value of < 0.05 was considered statistically significant.

Chronic kidney disease patients on dialysis were classified as above, below, or within the PTH target range of 2–9 times the upper limit of normal as recommended by the KDIGO guidelines, using the two PTH assays. A kappa statistic was used to assess the performance of the classification using both assays, with a value of ≥ 0.60 representing acceptable agreement.^24^

## Results

### Method comparison

A total of 505 residual patient samples were initially identified for the study. However, 24 samples were excluded: 17 had results exceeding the measuring range of the Elecsys PTH 1–84 assay and seven had exceeded the stability period. Thus, 481 samples were analysed and utilised for the method comparison study as shown in Online Supplementary Figure 5a.

**FIGURE 1:**
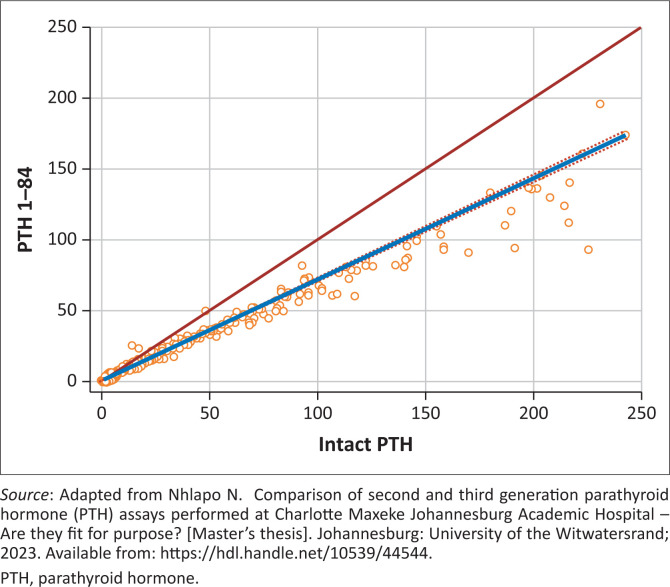
Distribution of results across all measured concentrations comparing intact parathyroid hormone and parathyroid hormone 1–84 assays (*y* = 0.713*x* + 0.887; *n* = 481), Johannesburg, South Africa, 06 April to 21 September 2022.

The median PTH concentration for the third-generation assay was lower, at 8.51 pmol/L (interquartile range: 4.30 – 25.5) compared to the second-generation median of 9.85 pmol/L (interquartile range: 4.53 – 33.58), and the difference was statistically significant (*p* < 0.0001). The regression analysis equation was calculated as *y* = 0.713*x* + 0.887 (Figure 1). There was good correlation between the two methods, with a coefficient correlation (*r*) = 0.994 and *p* < 0.0001. The intercept was calculated at 0.887 pmol/L (95% CI: 0.788 – 1.005) and the slope at 0.713 pmol/L (95% CI: 0.703 – 0.723), indicating that there were systematic and proportional differences between the two methods.

**FIGURE 2:**
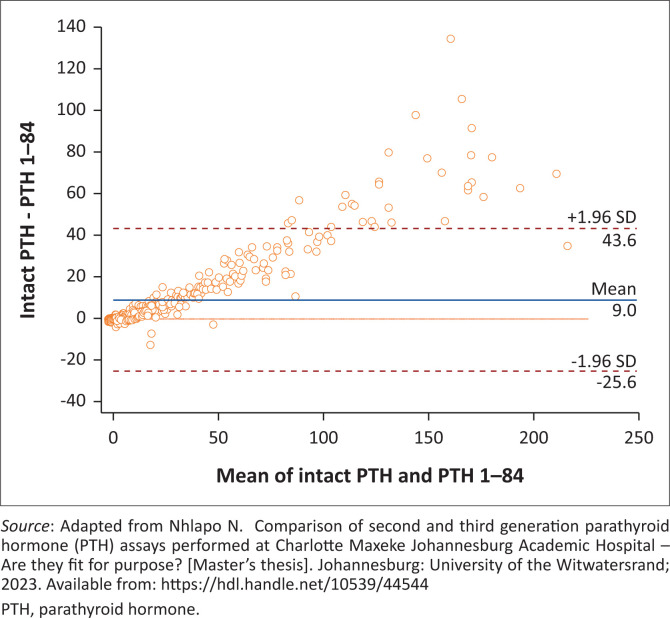
Absolute differences in parathyroid hormone (PTH) results across concentrations levels comparing intact PTH and PTH 1–84 assays, Johannesburg, South Africa, 06 April 2022 to 21 September 2022.

**FIGURE 3:**
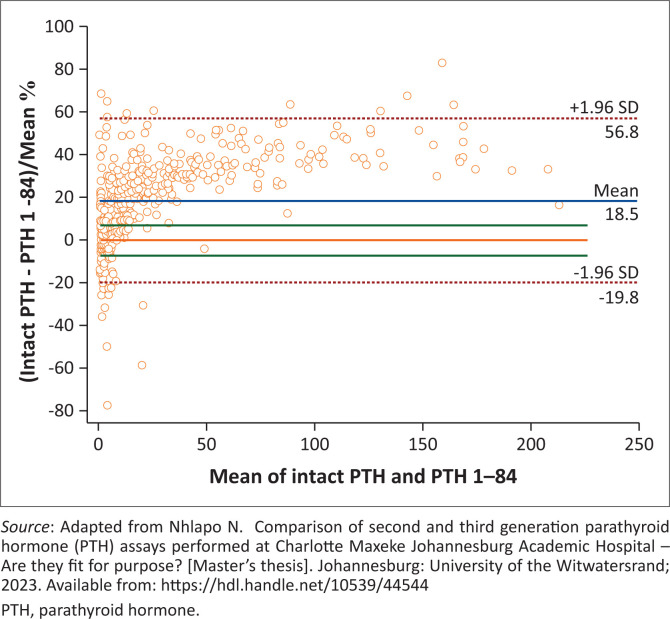
Percentage difference in parathyroid hormone (PTH) results across concentration levels comparing intact PTH and PTH 1–84 assays, Johannesburg, South Africa, 06 April 2022 to 21 September 2022.

The Bland-Altman analysis showed an average overall difference between the PTH assays of 9.00 pmol/L (18.5%, *p* < 0.0001) among all samples analysed, with increased deviations observed at higher PTH concentrations (Figure 2 and Figure 3). The bias in the entire CKD group was 27.2%, and 25.3% in the pre-dialysis CKD group (*p* < 0.0001). The dialysis group, however, demonstrated the highest bias of 35.2% (*p* < 0.0001). This is in stark contrast to the difference observed within the non-CKD group with estimated GFR (eGFR) > 60 mL/min/1.73 m^2^, which was demonstrated to be 5.7% (*p* < 0.0001) (Online Supplementary Figures 1–4).

### Clinical interpretation

The clinical performance of the two assays was assessed in the diagnosis of hypoparathyroidism and hyperparathyroidism and in CKD patients, including those on dialysis. The same 505 residual samples considered for the method comparison study were reviewed for the clinical interpretation arm of the study. Sixteen (16) were excluded because they exceeded the stability period and had missing demographics, 110 were from patients with an age less than 18 years old, and 103 had missing biochemical data. A total of 276 patients (170 women, 106 men, age range: 18 – 89 years) were included in the clinical interpretation part of the study as depicted in the flow diagram (Online Supplementary Figure 5b). The overall median age was 52 years with the majority being women (62%). The median age for CKD patients was lower than the non-CKD; however, the difference was not statistically significant (*p* = 0.1032) (Table 1). Vitamin D results were not included, as very few patients had results available on the laboratory information system.

The McNemar test did not demonstrate any statistically significant differences in clinical interpretation, as shown by *p*-values above 0.05 in the detection of low, normal, and high PTH using both assays. In both the CKD and dialysis group, no statistically significant (*p* = 0.500) differences in interpretation were found using McNemar, as is shown in Table 2.

**TABLE 2:** Differences in clinical interpretation using manufacturer reference interval, Johannesburg, South Africa, 06 April 2022 to 21 September 2022.

Parameter	Category	Intact PTH		PTH 1–84		*p*
		*n*	%	*n*	%	
Overall	PHPT	15	5.4	16	5.8	1.0000
	SHPT	185	66.5	184	66.2	1.0000
	Normal	57	20.5	58	20.9	1.0000
	Hypoparathyroidism	20	7.2	21	7.6	1.0000
CKD	SHPT	133	91.1	135	92.5	0.500
	Normal	11	7.5	9	6.2	0.500
	Hypoparathyroidism	2	1.4	2	1.4	UTC
Dialysis	SHPT	28	100.0	28	100.0	UTC

*Source*: Adapted from Nhlapo N. Comparison of second and third generation parathyroid hormone (PTH) assays performed at Charlotte Maxeke Johannesburg Academic Hospital – Are they fit for purpose? [Master’s thesis]. Johannesburg: University of the Witwatersrand; 2023. Available from: https://hdl.handle.net/10539/44544

PHPT, primary hyperparathyroidism; SHPT, secondary hyperparathyroidism; UTC, unable to calculate and perform test; PTH, parathyroid hormone; CKD, chronic kidney disease.

There were 29 patients with CKD on dialysis. Classification according to KDIGO guidelines revealed minimal discordance between the two PTH assays, with a weighted ҡ coefficient of 0.797 (0.594–1.000) (Table 3).

**TABLE 3:** Classification according to Kidney Disease: Improving Global Outcomes guidelines for intact parathyroid hormone and parathyroid hormone 1–84 assays, Johannesburg, South Africa, 06 April 2022 to 21 September 2022.

Manufacturer ULN (pmol/L)	< 2 × ULN		2–9 × ULN		> 9 × ULN	
	*n*	%	*n*	%	*n*	%
Intact PTH (1.6–6.9)	0	0.0	15	53.6	13	46.4
PTH 1–84 (1.6–6.0)	1	3.6	16	57.1	11	39.3

*Source*: Adapted from Nhlapo N. Comparison of second and third generation parathyroid hormone (PTH) assays performed at Charlotte Maxeke Johannesburg Academic Hospital – Are they fit for purpose? [Master’s thesis]. Johannesburg: University of the Witwatersrand; 2023. Available from: https://hdl.handle.net/10539/44544

ULN, upper limit of normal; PTH, parathyroid hormone.

## Discussion

The study conducted at a tertiary hospital in Johannesburg, South Africa, between 06 April 2022 and 21 September 2022 found that there was a difference (inter-assay bias) between the second- and third-generation PTH assays. However, there was no clinically significant change in the classification of hypoparathyroidism, primary hyperparathyroidism, and secondary hyperparathyroidism in pre-dialysis and dialysis CKD patients when one assay was used over the other.

With regard to analytical performance, the methods were strongly correlated. However, as the percentage mean bias surpassed the European Federation of Clinical Chemistry and Laboratory Medicine biological variation goal, this implies that these assays are neither interchangeable nor comparable. Our study showed that for the entire patient group, the third-generation PTH assay produced results that were, on average, approximately 20% lower than the second-generation assay, and even lower in CKD (27%) and dialysis (35%) patients. This is in keeping with other reported research.^3,14,15,25,26,27^ Studies by Hashim et al. conducted in a Malaysian population in 2020 and O’Flaherty et al. published in 2013 from the United Kingdom, both using Roche PTH assays, found that third-generation assays were lower by 30% and 40% compared to second-generation assays, respectively.^28,29^ In contrast, in the non-CKD group with eGFR above 60 mL/min/1.73 m^2^, the observed bias was 5.7%. This indicates that in patients with normal GFR, there are minimal differences between second- and third-generation assays, which is in keeping with other reported studies conducted in France and Malaysia.^26,28^

Although the observed differences between the two PTH assays were higher in both the pre-dialysis and dialysis CKD groups, there was no change in interpretation of secondary hyperparathyroidism with either assay. The KDIGO guidelines do not state the target PTH range in pre-dialysis CKD patients.^15^ Furthermore, we did not find studies that assessed the impact on clinical interpretation in this group.

There is a paucity of studies that have compared second- and third-generation PTH assays in dialysis patients using the KDIGO classification of 2–9 times the upper limit of normal.^26,30^ In the dialysis group, using the manufacturer’s upper limit of normal, classification using KDIGO did not demonstrate statistically significant discordance between the two assays. This study was limited by a small sample size of confirmed dialysis patients which reflects the limited dialysis resources in our country. However, our finding contrasted with a few published studies, including that by Dupuy et al. conducted in France and published in 2018, which demonstrated a high percentage of discordant results in dialysis patients, with a ҡ coefficient of < 0.20.^26^ In addition, a study by Beko et al., published in 2012 from Hungary, found that using the manufacturer’s upper limit of normal, the clinical classification was altered in up to 23% of dialysis patients because of the switch from second- to third-generation PTH assay.^30^ Presence of discordance is important, as PTH concentrations are used in the management of CKD-MBD. Hence, the use of second- over third-generation PTH assays may result in misclassification, leading to inadequate management.

There have been various attempts to reduce the discrepancies between the second- and third-generation PTH assays in dialysis patients, by applying a correction factor or deriving an equation to allow interconversion between the two assays.^31^ A study by Wójtowicz et al. conducted in 2016 in Poland suggested the use of 1–84/7–84 PTH ratio to report PTH results to minimise potential misdiagnosis of CKD-BMD in CKD patients.^31^ However, further validation studies are needed, and these efforts are not currently adopted by clinical guidelines.

Overestimation of PTH by second-generation assays because of PTH fragment cross reactivity is of negligible consequence in patients with primary hyperparathyroidism. Nonetheless, the question regarding whether third-generation PTH assays offer superior diagnostic efficacy remains. In our study, there were no differences in interpretation between the two assays with regard to diagnosis of primary hyperparathyroidism. This is consistent with other published studies: Boudou et al. from France,^32^ and Bonansea et al. from Brazil.^33^ Taking into consideration published evidence, the recent international guideline on evaluation and management of primary hyperparathyroidism recommend the use of either second- or third-generation PTH assays.^34^

The International Federation of Clinical Chemistry and Laboratory Medicine Committee for Bone Metabolism has established a working group for the purpose of creating PTH standardisation to make the results comparable among different assays.^35^ In the absence of a reference measurement method, the International Federation of Clinical Chemistry and Laboratory Medicine has recommended the use of the World Health Organization standard, but this has not been completely implemented because of a lack of commutability.^9,10^ Even though second-generation PTH assays are the most utilised assays, third-generation PTH assays offer the best hope for harmonisation of PTH because the majority are traceable to the World Health Organization standard.^35^

To the best of our knowledge, this is the first study in Africa to evaluate the differences in analytical performance and impact on clinical interpretation of second- and third-generation PTH assays.

### Limitations

Our study had some limitations. Firstly, we had a small sample size of patients on dialysis. Secondly, not all patients had laboratory results of tests used in the evaluation of CKD, for example urine albumin. Finally, the study was based on information available on the laboratory information system, with limited clinical information.

### Conclusion

The second- and third-generation PTH assays correlate very well, though there was a bias observed between the two methods which increases with rising PTH concentrations that prevail in CKD patients. However, using the manufacturer’s assay-specific reference intervals and KDIGO guideline on PTH classification, there were no major changes in clinical interpretation when either assay was used. Thus, the decision to use third- over second-generation PTH should take all of this into account. We recommend that assays not be used interchangeably. Therefore, as stated by the manufacturer and in accordance with the KDIGO recommendations, clinical laboratories should state the generation of PTH assay employed and inform clinicians of any change in clinical methods to facilitate appropriate interpretation of PTH results.
